# Plant-based diets for older adults in care homes: a realist synthesis

**DOI:** 10.1186/s12877-025-06927-0

**Published:** 2026-01-26

**Authors:** Saffron Whyton, Lisa Methven, Kathrin Cohen Kadosh, Praise-God Adedokun, Victoria Tischler

**Affiliations:** 1 School of Psychology, University of Surrey, Guildford, GU2 7YW England; 2 Department of Food and Nutritional Sciences, University of Reading, Reading, RG6 6UR England

**Keywords:** Plant based diet, Care homes, Older adults, Realist synthesis

## Abstract

**Background:**

Approximately 450,000 older people reside in UK care homes, which is expected to almost double within 20 years. 3% of the UK population follow a plant-based diet (absent in all animal foods), 13% of whom are aged over 65. Plant-based meals are not mandatory to be offered in care homes, however, providing these meals could positively affect health, and ensure dignity of choice for those who already follow a plant-based diet. This review aims to explore contexts, mechanisms and outcomes that could influence the success of a plant-based meal study.

**Methodology:**

A realist synthesis of the literature was used to develop initial programme theories. The stages of this synthesis was as follows: (1) Initial scoping. (2) Search for relevant evidence (3) Selection and appraisal of documents (4) Extract data. (5) Analysis and synthesis.

**Results:**

From 36 articles, eight initial programme theories were constructed, taking the form of context-intervention-mechanism-outcome configurations. Contexts identified included willing, open and motivated staff, residents who desire greater variety, and meals that are appetising, easy to consume and nutritionally adequate. Intervention activities included training for chefs, nutrition education for staff and tasting sessions for residents. Cross-cutting mechanisms used across the initial programme theories included increased physical opportunity and increased psychological capability. Outcomes either related to beliefs about or participation in the study or improvements to resident wellbeing.

**Conclusion:**

This realist synthesis addresses a gap in the literature relating to the provision of plant-based meals in care homes by exploring ways in which adherence and acceptability of plant-based meals can be enhanced amongst residents and staff. These findings will support the design of an empirical study where the gleaned initial programme theories will be tested *in vivo.* Additionally, the use of the realist synthesis presents a novel approach to designing complex nutrition interventions for specific contexts.

**Supplementary Information:**

The online version contains supplementary material available at 10.1186/s12877-025-06927-0.

## Background

Plant-based diets (PBDs) are defined as diets that eliminate all animal food products, including meat, eggs, dairy, fish, honey [1]. There is currently a lack of peer-reviewed data on adherence to PBDs across different populations, however, over the past two decades, the prevalence of the UK population who report following a PBD has grown, estimated to be between two to three percent [2]. Although commonly thought of as a diet adhered to by younger generations, a 2016 survey commissioned by the Vegan Society found 13% of those consuming a PBD are over 65 years old [3]. As of the most recent census, approximately 270,000 older adults reside in care homes (CHs) in the UK [4] - this is expected to almost double by the year 2050 [5]. A report commissioned by Vegetarian for life surveyed 1000 CHs and found that the number of residents following a PBD was estimated to have risen by 127% since 2014, with nearly 1% of the CH population predicted to follow a PBD by 2031 [6]. Due to a lack of knowledge amongst care staff surrounding PBDs, several instances have been identified by the vegetarian all-party parliamentary group where staff, either intentionally or unintentionally, provided those who follow a PBD with meals containing meat and fish [7]. 

The reasons for choosing a PBD includes religious or cultural restrictions (including Hindus, Buddhists and Jains), ethical and environmental concerns, lactose intolerance or egg allergy, hedonic preferences for PB foods, or for potential health benefits [8]. Specifically for older adults, PBDs can offer several potential health benefits. For example, data from the UK biobank cohort suggests an association between 10-year adherence to a healthy PBD and lower risk for dementia and depression [9]. These beneficial effects could be attributed to the high fruit, vegetable and wholegrain content of the diet [10]. Within the context of CHs, consumption of fruit, vegetables and wholegrains tends to be inadequate, as demonstrated by a recent European cross-sectional study [11]. Improving the intake of these foods could have several benefits on a resident’s health, for example, data from a UK survey of 227 CH residents found 45% had essential hypertension [12], which could be positively impacted by a diet higher in fibre [13]. Additionally, polypharmacy was also found to be highly prevalent [12], which could also be positively influenced by a PBD [14]. 

Although nutritional intake is an important consideration, CH mealtimes have a deeper psychosocial meaning for residents. Often cited as the most important time of day by both staff and residents, mealtime provides an opportunity for residents to establish social connections whilst also adding structure to their day [15]. Despite this importance, a resident’s view of mealtime can be negatively impacted by a lack of control over menu options and limited choice [16]. This is because autonomy is highly valued by residents [15]. Limited choice is a particular concern for those with specific dietary preferences, as meal alternatives are often not provided [7] or the alternatives provided lack variety [16]. This is despite Regulation 14 from the Health and Social Care Act 2008, which states ‘nutrition and hydration needs means the meeting of any reasonable requirements of a service user for food and hydration arising from the service user’s preferences or their religious or cultural background’ [17]. Given the high prevalence of dementia in CHs (70% prevalence in the UK) [18], it is also important to provide options that meet previous dietary preferences, so staff can uphold their dietary beliefs on the resident’s behalf. 

Considering the value of mealtimes, it is important to consider how food provision can be improved to ensure preferences can be met. A range of different interventions have been trialled in CHs to improve meal provision [19]. Whilst altering the method of food service and the dining environment can bring some benefits [19, 20], interventions with the most enduring, impactful outcomes are those that provide more or higher quality food [19, 21]. Providing a PB option in CHs therefore is beneficial in multiple respects: improving food-related quality of life, enhancing dignity of choice and autonomy, and potentially boosting nutritional intake.

This review forms the first stage of the PLANT-CARE programme of research. The overarching aim is to explore the opportunities and challenges for providing PB meals in CHs. The overall purpose of this research is to ensure PB meals are provided as an extra choice, enabling those already following a PBD to be able to uphold their beliefs, and benefitting the health of omnivorous residents by providing greater dietary variety. A later stage of this research will include an empirical study across multiple CHs, where PB meals are provided to all residents alongside current menu offerings for a period of four weeks. The end result of this programme of research is to provide a succinct set of recommendations for policy with regard to how best supply PB meals in CHs.

To inform the design of the PLANT-CARE empirical study, the literature was systematically searched to answer the following research questions:

1. In what context will a PB meal study be successful?
2. What are the mechanisms by which we expect a PB meal study to elicit its associated outcomes?
3. What are the potential outcomes that could be elicited by a PB meal study?

## Methodology

This review follows the RAMESES (Realist and Meta-narrative Evidence Syntheses: Evolving Standards) guidelines for the reporting of realist reviews. The steps taken to search for and retrieve literature follows a previously proposed methodology (Table 1). The review protocol was submitted to PROSPERO (International Prospective Register of Systematic Reviews) to improve transparency (CRD42024600852) [22].

**Table 1:** Realist synthesis methodology

Step	Task
1. Clarify scope of the review	Generate initial theories via a consultation with the research team, using pre-existing knowledge of literature and by using substantive theories
2. Search for relevant evidence	Search electronic peer-reviewed databases using keywords and Medical Subject Headings (MeSH)
3. Selection and appraisal of documents	Use inclusion and exclusion criteria to screen for literature. Retrieve full text of relevant articles
4. Extract data	Extract general study data onto Excel spreadsheet. Search reference lists by hand for additional relevant literature. Also search for grey literature
5. Analysis and synthesis	Analyse data for context and outcome patterns; and synthesise mechanisms

Framework for the realist synthesis methodology proposed by Pawson et al., 2005 [23]. Definitions of realist terms can be found in additional file one

### Clarify scope of the review

**Figure. 1:**
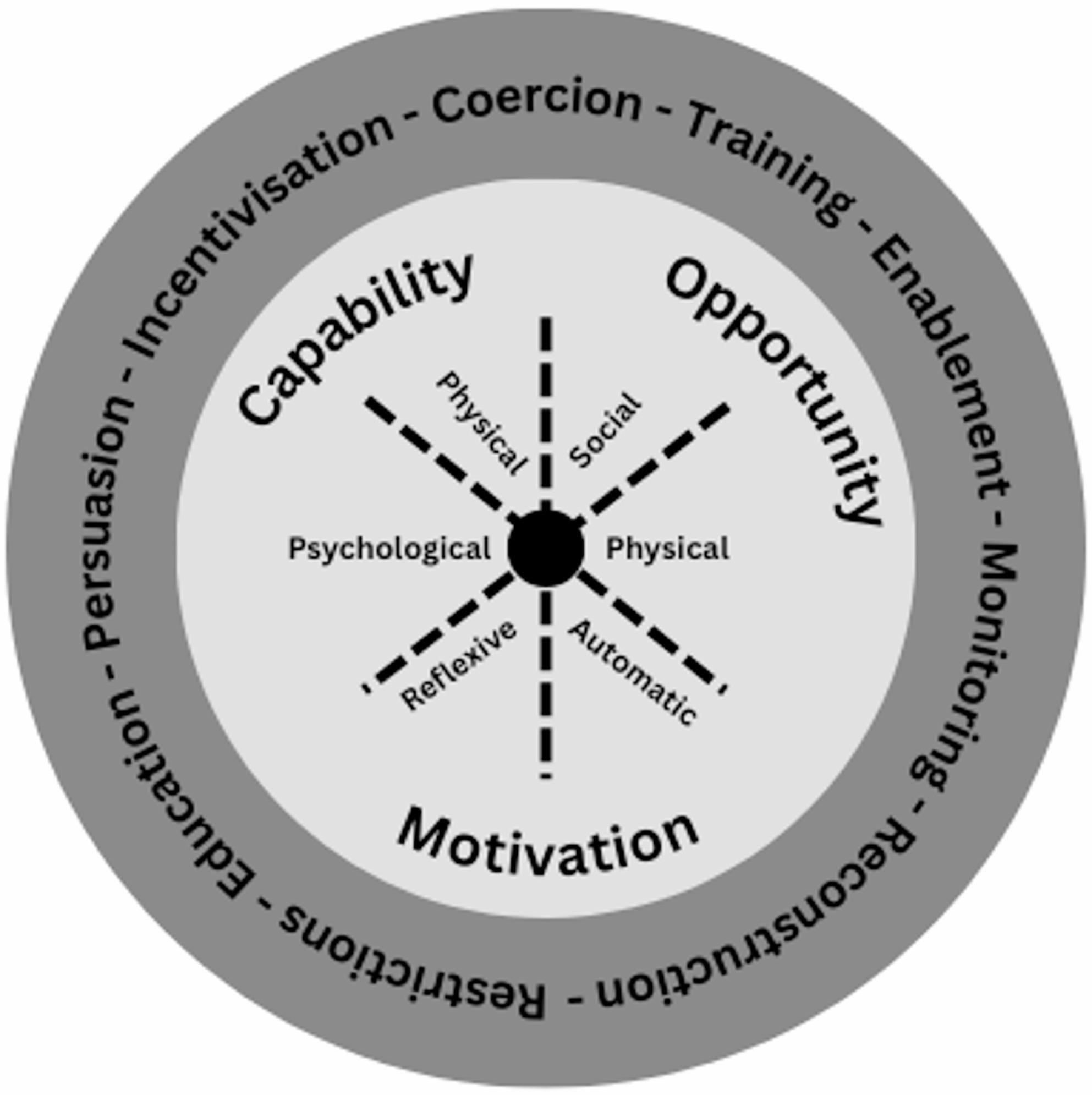
Behaviour change wheel: modified from Michie et al., 2011 [25]. The outer layer represents the intervention activities, whilst the inner layer represents behaviour change mechanisms

To clarify the scope of this realist synthesis, a substantive theory with sufficient explanatory power to contribute towards theory formation was identified. The chosen theory was the behaviour change wheel (Figure 1), which is rooted in the capability-opportunity-motivation-behaviour (COM-B) model. The COM-B model was expanded upon via a systematic review of 19 change frameworks to include behaviour change techniques and methods to target policy change [24]. The rationale for the use of the behaviour change wheel was three-fold: It has previously been used to help guide the design of interventions; it is adaptable to different contexts; and it links casual mechanisms with intervention activities [24]. The intervention activities identified in this model were used to guide the identification of relevant literature.

To help build search terms, an initial scoping of the literature was completed. This literature scoping alongside a consultation with the research team (SW, VT, KCK, LM) helped to glean a set of candidate theories (see additional file two). These will be refined into initial programme theories (IPTs) as a result of this realist synthesis. This scoping also identified two key articles that were used in the final evidence synthesis.

To further clarify the scope, key words were identified and defined to generate search terms. A CH is defined as ‘places where people live in later life to receive extra support with personal care, such as eating, washing, dressing and taking medication’ [25]. This can include the terms ‘residential homes’ and ‘nursing homes’ but not acute care facilities (skilled nursing facilities etc.), facilities providing specialised care (dementia care wards) or facilities that do not provide any level of care (retirement homes etc.).

Finally, the decision was made to not use stakeholders at this stage of the research. Instead, the current synthesis will inform a qualitative interview study with stakeholders. By taking a sequential approach, any primary data collection can be guided by the research gaps identified in this review [26].

### Search for relevant evidence

Due to a lack of evidence specifically on the topic of PBDs in CHs identified through the initial scoping of the literature, two searches were completed. Each search was conducted across five peer-reviewed databases, including clinicaltrials.gov, PsycINFO, Scopus and Web of science, as well as CINHL (Cumulative Index to Nursing and Allied Health Literature) (used for search one) and EMBASE (used for search two). For both searches, a mix of free-text and major headings were used. Pre-exclusion filters were applied: this included English-only, published between 2000 and 2025 and full text availability. The search was conducted in January 2025, with data extracted and analysed until April 2025. The search was then rerun on a single database in early April 2025 to check whether new relevant papers had been published. An automated deduplication tool was used, with the accuracy checked by the lead researcher (SW). Citation pearling (via backwards indexing) was employed on relevant articles to retrieve further evidence. Search one retrieved literature on food and nutrition in CHs, with a focus on qualitative research, as well as intervention trials that were comparable to the proposed PLANT-CARE empirical study. This search query was built using a Population-Concept-Context framework where population is a CH, the concept is nutrition/dietary interventions, and the context is the dining room: (Care Home*) OR (Long Term Care) AND (Nutrition*) OR (Diet*) AND (Dining) OR (Dinner). Search two retrieved relevant PB dietary interventions conducted in different contexts. A simple search query framework was used to identify the exposure of interest (PBDs) and the methodology (interventions, trials etc.): (Plant-based diet*) OR (Vegan) AND (Intervention*) OR (Trial*). The full search terms are listed in additional file three.

### Selection and appraisal of documents

Informed by the initial scoping of the literature, an inclusion and exclusion criteria was used to screen the retrieved studies (Table 2). For systematic reviews and meta-analyses that were relevant to the study, backwards indexing was employed to retrieve original data that could contribute to IPT formation.

**Table 2:** Inclusion and exclusion criteria

Inclusion	Exclusion
**CH Nutrition and Mealtime Studies**	
Qualitative and mixed methods studies	Studies without a qualitative element or psychosocial outcomes, systematic reviews/meta-analyses
Must be in a CH, nursing home or residential home	Not a hospital, dementia care ward, retirement home, acute care home
Interventions relevant to the context of this study	Meal interventions to modify resident behaviours (such as aggressive behaviour towards staff), malnutrition prevalence, oral nutritional supplements, texture-modified diets or dysphagia, meat or diary-based interventions and therapeutic diets (for example, diabetic).
**Plant-based Interventions**	
Any experimental design (such as randomised controlled trial, pilot trials, quasi-experimental)	Studies without a qualitative element or psychosocial outcomes, systematic reviews/meta-analyses, cross-sectional studies/case controls (as are not comparative to proposed PLANT-CARE intervention or the CH population)
Intervention must be focused entirely on diet	Studies with exercise, stress or other lifestyle interventions
Intervention must be a PBD	Mediterranean, portfolio diet, DASH, MIND or vegetarian diet [27]
Healthy adults (18 + years old), adults who are overweight	Do not include interventions specifically for those with specific non-communicable diseases such as cancer, arthritis etc. Studies in children and school settings

Full inclusion and exclusion criteria for both literature searches used for the screening of title and abstracts.

*DASH* Dietary Approaches to Stop Hypertension,* MIND* Mediterranean-DASH Intervention for Neurodegenerative Delay

Title and abstract screening took place between the lead researcher (SW), who analysed the entire data set, and a secondary researcher (PGA) who analysed 50% of the data set, ensuring the reliability of the screening criteria (Table 2). Any disputes were raised and resolved between the researchers. Full-text screening was then completed primarily by the lead researcher (SW) to analyse for relevance. 1% of the retrieved studies (*n* = 484) were analysed for relevance by a secondary researcher (PGA). Studies deemed relevant were checked for methodological rigour using the CASP (Critical Appraisal Skills Program) toolkit, as well as the JBI (Joanna Briggs Institute) appraisal toolkit for quasi-experimental studies. The full selection and screening process is detailed in a PRISMA flow diagram (see additional file four)*.*

### Data extraction, analysis and synthesis

Generic data (authors, date, experimental design, results etc.) was extracted into Excel (see additional file five.) Analysis of the literature took the following steps:

1. Full texts were inductively and deductively coded by one researcher (SW) using NVivo 14 (QSR International, Cambridge, MA, USA). A list of codes was created prior to analysis which were derived from the researcher’s current knowledge of the retrieved papers. New codes were added during analysis when evidence was found that did not already sit within pre-made codes.
2. Coded regions were copied into a word document and categorised based on the intervention activities they were in reference to (education, training etc.). For codes that did not align to an intervention activity, such as data retrieved from interview studies that weren’t in reference to an intervention, themes were created. These themes included ‘maintaining autonomy’, ‘personal preferences’ and ‘dining atmosphere’.
3. For themes that had a significant number of codes assigned, codes were further split into positive and negative sub themes. Codes were labelled positive if the contexts and mechanisms that were identified supported positive outcomes, whilst negative codes denoted contexts and mechanisms that did *not* support positive outcomes or generated negative outcomes.
4. The categories were then aligned to the candidate theories previously generated (see additional file two). New IPTs were created for data that did not support the candidate theories.
5. Within each group, the coded regions were further segmented into contexts (the interventions backdrop that influence the outcomes), mechanisms (how the intervention activity elicits outcomes) and outcomes (the effects produced by the interaction of contexts, intervention activities and outcomes.).

Outcomes and contexts were generally easy to identify, but most mechanisms were based on the behaviour change wheel. The mechanisms were chosen based on the intervention activity they were in reference to. To ensure the outcomes and contexts identified in the literature were adequately supported, demi-regularities (semi predictable patterns) that occurred multiple times across different papers were chosen for the final IPTs. Furthermore, demi-regularities had to occur in papers retrieved from both search one and search two.

## Results

In total, eight CIMOCs (Context-Intervention Activity-Mechanism-Outcome Configurations) were formed from the candidate theories and the literature search.

### Study characteristics

Overall, 5111 records were retrieved and analysed, including those retrieved through backwards indexing (see additional file four for full details). After title and abstract screening, 331 articles were retrieved from search one (interventions in CHs) and 153 articles from search two (PB interventions). Full texts were then analysed for relevance to the PLANT-CARE programme of research. 36 articles were kept and used for theory generation – 21 were from search one, 13 were from search two, and two were identified from the initial literature scoping. Study designs included 23 qualitative (surveys, interviews, ethnography etc.), 12 experimental (including RCTs, quasi-experimental, pilot trials etc.) and one grey literature article. Quality assessments revealed five of the retrieved studies were of poor methodological quality, 18 were of moderate quality and 11 were of high quality (full details see additional file five)*.*

Table 3 aligns the eight CIMOCs with the corresponding stage of the PLANT-CARE empirical study. The stages of the study were formulated based on the findings from this review. The findings suggest a wide variety of intervention activities (meal preparation training, nutrition education etc.) are needed to incorporate all actors involved in the empirical study (chefs, staff, residents). Furthermore, certain established contexts are necessary to facilitate successful incorporation of PB meals, including willing and motivated staff, chefs who understand the wider importance of providing PB meals to residents, and a CH structure that facilitates inter-professional collaboration. Due to the different actors involved in a CH study, it was necessary to ensure that the views of all these actors were explored: six studies focused on care staff [30, 31, 37, 39, 40, 56], one focused on chefs [38], one focused on family members [50] and eight studies focused on residents [16, 28, 41, 47, 51, 54, 55, 57].

**Table 3:** Ordered initial programme theories

Phase of empirical study	Initial programme theories
Understanding the context	CIMOC one: Budgetary and time constraints of care homes
Upskilling the staff	CIMOC two: Meal preparation training to enhance preparation confidence and create sensorially appetising meals
	CIMOC three: Plant-based nutrition education for staff to improve adherence to the empirical study
Creating the meals	CIMOC four: Collaboration between staff and nutritionist during meal design
	CIMOC five: Meeting the physiological and nutritional needs of residents
	CIMOC six: Ensuring meal familiarity to enhance resident satisfaction
	CIMOC seven: Resident feedback on meals to enhance autonomy
Providing the meals	CIMOC eight: Previous familiarity or desire for choice

Contexts-Intervention Activity-Mechanism-Outcome Configurations (CIMOC) developed through literature search aligned to their proposed stage of a plant-based meal study

All theories are supported by the behaviour change wheel with the intervention activities matched with the corresponding behaviour mechanism of the COM-B model. Table 4 showcases the IPTs in full, using an ‘if-then-because’ framework with literature and quotes to support. Further information on how theories were generated using the selected literature can be found in *additional file six*. Four of the eight CIMOCs (CIMOC two, three, four and five) were expanded versions of candidate theories (*additional file two*) generated through initial literature scoping and a research team meeting. The remaining CIMOCs were gleaned based on findings in the literature. All aspects of each IPT were supported by literature, with abstractions made where needed. To demonstrate an example abstraction, the intervention activity for CIMOC eight is providing a PB option, however the literature supporting this theory is mainly rooted in interventions where more choice is added into a CH [41, 49, 57].

**Table 4:** Expanded initial programme theories

Initial programme theory	Quotes to support
*CIMOC one: If CHs that are limited by budgetary and time constraints *[28–31] *(Context) are provided with affordable*,* healthy PB swaps *[30, 32–35]* that are easy to prepare *[36]* and are considerate the CHs needs* [30, 37]* (Intervention Activity)*,* then acceptance *[33]* and adherence *[34, 35]* to the requirements of the empirical study will increase (Outcome) because the staff are more likely to perceive the PB meals positively *[34, 35]* (Mechanism)*,* increasing perceived psychological capability *[33, 34, 36, 37]* (Mechanism)*	“We weren’t given any extra time for the extra work… [name of cook] did a lot of work out of hours at home and never got reimbursed” [39]. “The costing too…—fresh fruits and vegetables—everyone wants that, and so it’s nice to have that. It’s a lot of work for the staff to prepare it and, nutritionally, you can still get quite a bit of nutrition from frozen and in some cases canned” [30].
*CIMOC two: If chefs*,* who are motivated to provide healthy meals and are interested trying new menu options* [36, 38, 39]* (C)*,* are provided with training as to how to cook and prepare appetising PB meals *[38, 40–43]* (IA)*,* then chefs are more likely to accept the requirements of the empirical study (O)*,* because of improved perceptions of the PB meals *[38],* and the meals provided will be sensorially appetising *[38]* (O)*,* because of increased physical capability *[38, 40]* (M)*,* which will encourage residents consumption *[21, 37, 38]	“The biggest problem is motivating other staff members to see why it is a good idea to make changes” [38]. “It is all so rusted shut, new ideas had to be brought in. Some cooks have been working here for 100 years, but we also have a few guys who really enjoy trying something different” [40]. “I feel the other thing is the lack of skilled workforce in foodservice. . there’s just no training from the foodservice to the carers to the table really” [37]. “Presenting the meal that is appealing to the eye … that is important because I am a picky eater” [53] “…what was wrong with the way they were made before? Are we not doing our job properly here? …I just feel as if you’re being undermined somehow.” [39]
*CIMOC three: If staff*,* who are willing to change their perceptions of PBD *[39, 40]* (C) value providing nutritious foods to residents (C) and anticipate a positive reaction from residents (C)*,* are provided with PB nutrition education *[31, 33–37, 40, 44–47]* delivered by a nutritionist *[37, 48]* (IA) then staff will be more likely to adhere to the requirements of the empirical study and support the PB meal delivery *[31, 44]* (O)*,* because their perceptions of PBDs will become positive *[34, 36, 46]* (M)*,* making them more reflexively motivated to provide PB meals *[48]* (M) and their trust will improve due to involvement of nutritionist *[48]* (M)*	“We have the opportunity to impact on every resident’s life six times a day. . by providing them nutritious, healthy, beautiful looking tasty food” [37]. “I think that a lot of times in aged care the short-term cost of training and getting people and all that, they don’t see the long-term benefits, if that makes sense” [37]. “Culture and attitudes, biggest barrier. Especially if you’ve got a working site where they’ve had a lot of staff there for a long period of time. But you can make the change, You’ve just got to chip away…” [37]. “Try something new like couscous or bulgur. That is possible. Maybe they [the residents] really like it, but if we do not serve it to them, we do not know” [37]
*CIMOC four: If all care staff*,* who are motivated to foster change *[34, 37, 39]* (C) and the structure of the CH allows collaboration *[37, 39, 41]* (C)*,* engage in meal development activities together *[16, 50]* (IA)*,* then they will be more likely to adhere to the intervention *[39]* (O)*,* because they will feel greater ownership over the meals *[39]* (M) and their confidence in the intervention will improve *[37, 39]* (M)*	“Why are kitchen staff not actively involved decisions made by facility management?” [40]. “Probably lack of leadership in people higher up in management driving that change, because I think without their support it’s very hard for foodservices to make a change” [37]. “I think everyone should be advocating. I think it’s again—… it’s a case of communication. So everybody should be effectively trying to achieve that goal…” [37]
*CIMOC five: If meals are designed to meet the physiological and nutritional needs of residents *[29, 51]* (C) by the use of easy to chew*,* high protein meat-alternatives (IA) delivered by trained chefs *[38]* (IA) then residents will be more likely to enjoy the meals *[47, 51, 52]* (O)*,* leading to increased consumption (O)*,* because they will have greater perceived physical capability *[52]* (M) and improved reflexive motivation *[52]* (M)*	“[If I eat] meat that is a little bit more fatty, … I’m going to feel heavy… it seems like all my energy is focused on digestion. Whereas, if I have a legume-based meal,…I feel better… the process is less demanding on my body, my energy” [52].
*CIMOC six: If meals are designed to be culturally appropriate*,* familiar and traditional *[28, 30, 33, 51, 53]* (C)*,* via the use of health PB swaps and cultural staples *[36, 39, 42, 47, 50]* (IA)*,* then residents will be more likely to accept and choose the PB option *[51]* (O)*,* because they will be automatically motivation to choose it *[32, 37, 47, 53]* (M) as it will be perceived as safe and comforting *[42, 51]* (M)*	“I did a lot of recipes that were familiar…and I would turn them into healthier versions of their favourite recipe… I believe, that was well accepted” [33]. “My residents would like to eat beans, potatoes and cabbage all the time. That’s the fact of our location.” [56] “I said to them, in a nice way, ‘Look, I don’t eat nothing else but English food and I’m not going to start it, I’m sorry’” [28]. “We’re not used to it. We don’t have the reflex to grab [and eat PB protein]” [52].
*CIMOC seven: If residents desire greater autonomy and choice over food options *[37, 54, 55]* (C)*,* are given the opportunity to provide feedback on PB meals during a food tasting session *[16, 28, 29, 37, 41, 49, 56–58]* (IA)*,* then their wellbeing will improve *[16, 54, 57]* (O) because they will feel empowered *[41]* as they have had increased physical opportunity to exercise autonomy *[37, 41, 54, 56, 59]* (M)*	“Resident involvement in choices was not always possible, and certain restrictions, such as dietary requirements, reduced the successful implementation of choice” [16]. “We organize a meeting with the residents once a week, so they can tell us what they want to eat” [56]. “This would help residents feel like they are able to participate in the operation of their home and that their contribution is valued by staff” [37]. “But most aged care facilities I’ve noticed are going to the food forums. They’re asking the residents. It’s all about the new standards as well, because it’s all about choice, so what is it you want” [37].
*CIMOC eight: If residents*,* who are familiar with PB foods *[36, 44, 52, 60]* or are open to or desire new options *[35, 44, 52, 59]* (C)*,* are provided with a new PB choice that they are free to choose *[28, 32, 34, 37, 41, 45, 49, 54, 59, 61]* (IA)*,* then they will be more likely to consume the PB option *[34, 37, 49]* (O)*,* because of improved physical opportunity to choose a PB option *[43, 52]* (M) and they will have improved wellbeing *[54, 59]* (O)*,* because of the additional opportunity to exercise autonomy over their meals *[41, 54, 56, 59]	“Being in a multicultural home makes it difficult to meet all residents’ preferences… Some East Indian vegetarians don’t like what European vegetarians like” [29]. “I have eaten [PB meals] a few times and they were good, so it’s encouraging to continue the experience” [52] “I object to the restrictions; we surely are capable of making a free choice” [55]. “…they might offer you two things…if you don’t like that or you don’t like that there’s nothing else” [16].

Column one shows the full initial programme theory using the heuristic context-intervention activity-mechanism-outcome configuration (CIMOC) tool. In the second column, supporting quotes from the literature have been presented

*PB* Plant-based, *C* Context, *M* Mechanism, *O* Outcome, *IA* Intervention Acitivity, *CH* Care Home

## Discussion

The purpose of this realist synthesis was to inform the design of an empirical study where PB meals are introduced to residents in CHs. This aims to ensure that residents have the dignity of choice to uphold previously held dietary beliefs or have the option to try new meals that could potentially offer health benefits. This synthesis explores the contexts, mechanisms and outcomes that inform a planned PLANT-CARE empirical study. 

The IPTs support defining success as the extent to which PB meals can be incorporated in CHs. The potential anticipated outcomes identified through this synthesis that could contribute to defining success include the acceptance of [33] and adherence to the empirical study [34] by key actors. A further anticipated outcome that could define the success of the PLANT-CARE study is the consumption of the meals by residents [49]. Anticipated secondary outcomes that could be triggered by PLANT-CARE study include improved resident wellbeing [54] and autonomy [41, 54]. Although not considered to define the success of the PLANT-CARE study, these secondary outcomes are likely to be influenced by the intervention activities and therefore will be outcome measurements gathered during the empirical study. 

This synthesis identified several key actors (residents, care staff and chefs) who need to be considered to ensure the empirical study is successful. By using individual intervention activities to target actors separately, CH-wide acceptance of the PB meals can be maximised. The activities identified include meal tasting sessions with residents; PB meal preparation training; nutrition education for staff; meal development where all CH staff work with a nutritionist to build the meals; swaps for PB ingredients. These intervention activities should help to trigger key mechanisms that help to improve adherence to and acceptance of the empirical study. 

Several key contexts were identified that could influence the success of the PLANT-CARE study. Staff adherence was identified as a key barrier to a study’s success [40]. The key context considered that would influence staff adherence was their mindset, specifically, their openness and willingness to change. Additional micro-level contexts included the communication between different professionals within the CHs, as well as the characteristics of the PB meals that could facilitate consumption. The only context considered beyond the micro-level was the structure of the CH, specifically with reference to employment hierarchies. It has been identified in previous research that contexts beyond the micro-level are harder to identify within literature [62] as they are often not reported on. This limits the extent to which all influencing contexts can be identified.

Due to a lack of retroductive theorising employed by authors in the papers retrieved, the identification of causal mechanisms was limited, with most mechanisms inferred via theoretical transferability using the behaviour change wheel. For example, where an intervention identified in the literature used education to increase intervention adherence, the corresponding mechanism as proposed by the behaviour change wheel would be increased psychological capability. Behavioural mechanisms are often not unique to specific settings and therefore can be abstracted and applied across different contexts [63]. Several mechanisms were gleaned from the literature however, such as improved perceptions of a PBD and increased trust. 

Although rapid growth in PB research has been seen in the previous decade [64], this field of research tends to be quantitative and health-focused, specifically using cohort and cross-sectional methodologies. This means that the interpretative qualitative research, where the views of stakeholders who consume PBDs or participate in PB interventions, is overlooked. The target population for many PB interventions are those with complex illnesses (such as cancer, arthritis, heart disease) or those who are overweight or obese. This limits the level of evidence that is applicable in this realist synthesis. Due to this lack of supporting evidence, several studies were included in this review that had low relevance to the proposed PLANT-CARE empirical study (see additional file six). The rationale for their inclusion is that they contributed to theory formation, particularly with a focus on identifying mechanisms and outcomes. The decision to use PB research that was not context-aligned came from the gap in the literature identified through the initial scoping. Although studies were identified through this synthesis that focused on improving CH mealtimes, much of the research in CHs focuses on improvements to health, specifically with regard to the ‘geriatric giants’ – namely incontinence, incompetence, immobility, impaired homeostasis and iatrogenic disorders [65]. The focus on physiological and psychological health means issues relating to sociocultural factors are neglected in research [65]. Therefore, the impact of provision of different dietary options has not been previously explored. Whilst using studies that did not align to the context of CHs proved useful in forming the IPTs, it does limit the strength of the conclusions. In order to confirm the IPTs, the gap in the literature on the topic of PBDs in CHs needs to be addressed. These gaps include residents’ acceptance of PB meals, the perceived feasibility from the perspective of staff on adding PB meals to menus, and the extent to which a PBD can support a resident’s health. The PLANT-CARE programme of research will aim to resolve these gaps.

Although realist research has been predominantly conducted for social research, its use for health-based research is increasingly prevalent [66]. Although PBDs have been promoted for their potential health benefits [67], there are considerations needed when providing these meals to CH residents. Elimination of animal foods from the diets of older adults could risk protein-energy malnutrition, particularly given the lower bioavailability and functionality of PB proteins [68]. Although some plant proteins could help support nutritional status [69] and muscle mass [70, 71], given the high prevalence of malnutrition in CHs [72], caution is needed when providing PB meals for CH residents. Carefully balanced PB nutritional support can be beneficial for treating malnutrition in the form of oral nutritional supplements [73], but no studies, to our knowledge, have prescribed PBD for malnourished older adults. Due to these potential limitations, health-related outcomes were not considered in the CIMOCs. Although a considerable amount of research is needed to address this gap, the PLANT-CARE empirical study will begin to populate this novel research area.

### Strengths and limitations

A key strength of this review is the diversity of professions in the research team, including nutritionists, psychologists and food scientists. A cross-disciplinary approach helps to reduce bias by incorporating multiple perspectives in the generation of theories. Although its limitations will be explored, the realist synthesis approach to building complex interventions will be a key strength of the future empirical research. Although still relatively novel, a realist synthesis goes into greater depth than a systematic review, with findings compiled from a variety of data sources [66]. This adds to richness and depth of the theories formed [74]. Using theory to inform complex interventions is recommended by the Medical Research Council [75]. Retroductive logic, which is commonly employed in realist research, allows a researcher to consider the complexities of causality [74]. Unlike typical positivist research which generally focuses on understanding what works, realist research goes beyond this boundary, considering why an intervention works, for whom and under what circumstances. Retroductive logic therefore is an additional strength of this synthesis, as it does not fall victim to an epistemic fallacy where it is inferred that evidence is generalisable across all contexts [66]. Furthermore, by simply taking surface level observations, positivist research fails to see the underlying mechanisms that trigger outcomes. This lack of consideration for both contexts and mechanisms can limit the success of a complex intervention.

The key limitation with this research is the methodological challenges that come with the use of realist syntheses. Despite the RAMESES reporting guidelines for realist synthesis, the methodological requirements to perform a realist synthesis are still ambiguous, with a set of principles used instead of an established framework [23]. This lack of standardisation suggests a realist synthesis lacks the rigour of a standard systematic review. Although the methodological flexibility may be practical to ensure a wide range of research areas can employ a realist approach, a lack of standardisation means findings may lack inter-individual reproducibility and papers may fall victim to suboptimal analysis [66]. Further to this, realist syntheses are partially rooted in subjectivity, meaning the researcher’s own interpretation of the evidence helped generate the IPTs. This could lead to overstating on both the impact of the intervention activities and the emergence of potential outcomes. This is also true for the underlying generative mechanisms identified, as they were largely abstracted from the behaviour change wheel due to a lack of retroduction employed in the studies retrieved. To overcome the subjective basis on which the IPTs have been formed, the next stage of research will involve formal consultation with stakeholders, which will enable refinement of the IPTs based on the views of those with expertise in CHs. 

Further, the use of non-context aligned studies (such as those centered in hospitals or workplaces) means the IPTs remain in the middle-range, suggesting that despite the abstractions made to apply them to the context of CHs, the IPTs still lack a degree of specificity to the CH context. The external validity of the PB studies retrieved has not been assessed by their respective authors, therefore suggesting the findings may not be applicable in other contexts. Despite this barrier, research exists on the opinions of both residents and care staff on food provision, which enabled inferences to be made about how mealtime-based interventions will be perceived. In realist research, it is often necessary to attempt to identify homogeneity in the form of demi-regularities, even in a collection of empirical studies that are highly heterogeneous [66]. 

Five of the 36 studies were deemed low quality based on the critical appraisal checklists used. The decision not to exclude these studies were based on their relevance to the formation of IPTs. It has been suggested by prominent researchers in the field of realist methodology that including a range of studies, regardless of quality, can help enhance the richness of the IPTs [23]. Despite this, the use of low-quality studies means the findings derived in this realist synthesis may lack validity. Further in vivo testing is required to support the IPTs generated from this research. A further limitation is on the scope of the CIMOCs. Due to high heterogeneity of UK CHs, methods of food preparation, staffing levels and financial circumstances may vary – this could infer several of the CIMOCs are not applicable to all CHs. At this stage of the PLANT-CARE programme of research, the IPTs are purely theoretical as there is still a degree of abstraction involved in their creation. The IPTs will need to be further refined and consolidated based on the CHs recruited for the empirical research. 

A final limitation is geographical variability where which the literature is derived. The PLANT-CARE study will take place in the UK. With limited evidence in a UK-only context, additional literature was drawn upon from a range of different locations (see additional file five.) Due to contextual differences, such as culture, policy and practices, both qualitative and quantitative evidence collected may not be applicable to a UK CH setting. The implication of this is that the IPTs gleaned from the results lack generalisable and specificity to a UK context. Further refinement is needed to take the IPTs from the middle range and tailor them to a UK context. Despite the limitations explored, this realist synthesis has allowed for the creation of IPTs which will be used to inform the design and delivery of a multi-component, theory-informed empirical study.

## Conclusion

This study has identified eight CIMOCs which will guide future research, including the design of a PBD study for older people in CHs. Future research within the PLANT-CARE programme of research, such as a stakeholder consultation and an empirical study, will help to refine and consolidate the CIMOCs. Beyond this, the research has wider significance for mealtimes in CHs. Several of the CIMOCs could help to inform improvements in nutritional care, for example, the importance of providing tasting sessions for new meals, or using cultural staples to enhance meal acceptability. Additionally, this study showcases a realist synthesis as a promising approach to designing nutrition interventions for specific contexts.

## Supplementary Information

Additional file 1. Definitions of key realist terms: realist terms used within main manuscript defined using established definitions.

Additional file 2. Candidate theories: initial programme theories generated from collaborative workshop with research team.

Additional file 3. Search Terms: Search terms used in databases with a mix of free text and major headings used across 5 databases for each search.

Additional file 4. PRISMA flow diagram: PRISMA flow diagram denoting the process of screening and the sources of literature.

Additional file 5. Study set information: generic information on the studies used in this realist synthesis.

Additional file 6. Building of theories: process of building initial programme theories from retrieved literature.

## Data Availability

Additional information on the data and materials used for this realist synthesis has been reported in the additional files provided and in the PROSPERO register.

## Abbreviations

PBD: Plant-based diet
PB: Plant-based
CH: Care home
IPT: Initial programme theory
CIMOC: Context-intervention activity-mechanism-outcome-configuration
