# Supplementary material for: Plant-based diets for older adults in care homes: a realist synthesis
Source: BMC Geriatr. 2026 Jan 26;26:233. doi: 10.1186/s12877-025-06927-0 (PMC12918464; doi:10.1186/s12877-025-06927-0)
Supplement: Supplementary file 1 — Additional file 1. Definitions of key realist terms: realist terms used within main manuscript defined using established definitions. [file 12877_2025_6927_MOESM1_ESM.docx]

| **Term** | **Definition** |
| --- | --- |
| CIMOC | A realist theory that links together the context, intervention activities, mechanism and outcomes, demonstrating the casual connection between them. |
| Context | Any condition that has existed prior to intervention. This includes any aspect of the local or wider environment, but also the participants’ circumstances. |
| Demi-regularities | Semi-predictable, reoccurring patterns or tendencies, observed in social phenomena, that are context-dependant and mechanism-driven |
| Mechanism | Mechanisms provide a casual pathway for how an intervention can led to an outcome, with the mediation of context. Reasoning mechanisms are unseen forces that comprise changes to a participants’ reasoning (cognitive or affective), attitudes and choice-making or in their capacity (skills, information, perspectives) Resource mechanisms are the components of an intervention that affect the outcome. |
| Outcome | Outcomes can be intended or unintended, resulting from an intervention. Desired process outcomes are considered as the prerequisites for a successful intervention. |
| Substantive Theory | Existing theory in particular disciplines that can be used to help understand interventions. |
