# Supplementary material for: Plant-based diets for older adults in care homes: a realist synthesis
Source: BMC Geriatr. 2026 Jan 26;26:233. doi: 10.1186/s12877-025-06927-0 (PMC12918464; doi:10.1186/s12877-025-06927-0)
Supplement: Supplementary file 2 — Additional file 2. Candidate theories: initial programme theories generated from collaborative workshop with research team. [file 12877_2025_6927_MOESM2_ESM.docx]

Candidate theories

| **Contexts** | **Mechanism** | **Outcomes** |
| --- | --- | --- |
| Supportive environment | Social opportunity | Increase likelihood to consume meals |
| Staff who are willing to accept intervention | PBD nutrition education | Increased intervention adherence |
| Staff who understand PBD benefits | Nudge residents’ consumption of meals | Improved fibre intake, decreased laxative reliance, increases risk of incontinence |
| Chefs who are keen to provide delicious meals | Provided with PB meal preparation training | Provide sensorially appetising meals, increased resident consumption |
| Staff who are concerned about overconsumption of meat in care homes/If beliefs about meat consumption cannot be changes | Provided with resources to provide residents with healthier alternatives | Increased encouragement to persuade residents to consume/Will not adhere to intervention |
| PB provided alternatives are easier to swallow | Improved physical capability | Increased consumption, improved mini-nutritional assessment (MNA) score |
| If PB meals provided are familiar to the residents | Through exposure activities, through perception of plant-based meat alternatives | Increased likelihood to be picked, may not meet expectations – won’t be chosen again, those with dementia may not recognise meal |
