# Supplementary material for: Plant-based diets for older adults in care homes: a realist synthesis
Source: BMC Geriatr. 2026 Jan 26;26:233. doi: 10.1186/s12877-025-06927-0 (PMC12918464; doi:10.1186/s12877-025-06927-0)
Supplement: Supplementary file 3 — Additional file 3. Search Terms: Search terms used in databases with a mix of free text and major headings used across 5 databases for each search. [file 12877_2025_6927_MOESM3_ESM.docx]

| Population | Concept | Context |
| --- | --- | --- |
| “Nursing home*” (MH) (DE) | Intervention* | Meal* |
| Homes for the Aged (MeSH) | “Dietary intervention*” | Eating |
| “Care home*” | “Nutrition intervention*” | Food |
| “Aged Care” | Nutrition* | Dining |
| “Institutionali#ed elderly” | Intake* | Dinner |
| “Institutionali#ed older adult*” | “Food intake*” | “Dining room*” |
| “Residential aged care” | Liking | “Dining setting” |
| “Residential home*” | Satisfaction | “Dining environment*” |
| “Assisted living” | Enjoy* | “Dining ambi#nce” |
| “Residential facility*” (MeSH) (MH) | Preference* | “meal ambi#nce” |
| “Care facility*” | “Nutritional status” | “meal environment*” |
| “Long term care” | “Nutritional intervention*” | “Dining Atmosphere” |
| “Residential care” | “Food choice*” | Lunch* |
| “Old age home” | Health* |  |
| “Senior Hous*” | “Nutrition* program*” |  |
| “Assisted#living facilities” (MeSH) | “Dietary Program*” |  |
| “Residential care Institutions” (DE) | Diet* |  |
| “Assisted#living elderly” | “Dietary management” |  |
| “Rest home*” |  |  |

Search 1 – Care home nutritional interventions

| Intervention | Methodology |
| --- | --- |
| Plant-based | Intervention* |
| ‘Plant-based food*’ | Pilot* |
| ‘Plant-based diet*’ (/exp/mj) (MeSH) (MH) | Intergrat* |
| ‘Plant-based nutrition’ | Program* |
| Vegan* | Trial* |
| ‘Vegan diet*’ (MeSH) (/exp/mj) | Educat* |
| ‘Plant-based protein*’ | Implement* |
| ‘Plant-based meal*’ | Experiment* |
| ‘Plant-based eating’ |  |
| ‘Plant protein*’ |  |

Search 2 – Plant-based interventions
