# Supplementary material for: Plant-based diets for older adults in care homes: a realist synthesis
Source: BMC Geriatr. 2026 Jan 26;26:233. doi: 10.1186/s12877-025-06927-0 (PMC12918464; doi:10.1186/s12877-025-06927-0)
Supplement: Supplementary file 4 — Additional file 4. PRISMA flow diagram: PRISMA flow diagram denoting the process of screening and the sources of literature. [file 12877_2025_6927_MOESM4_ESM.docx]

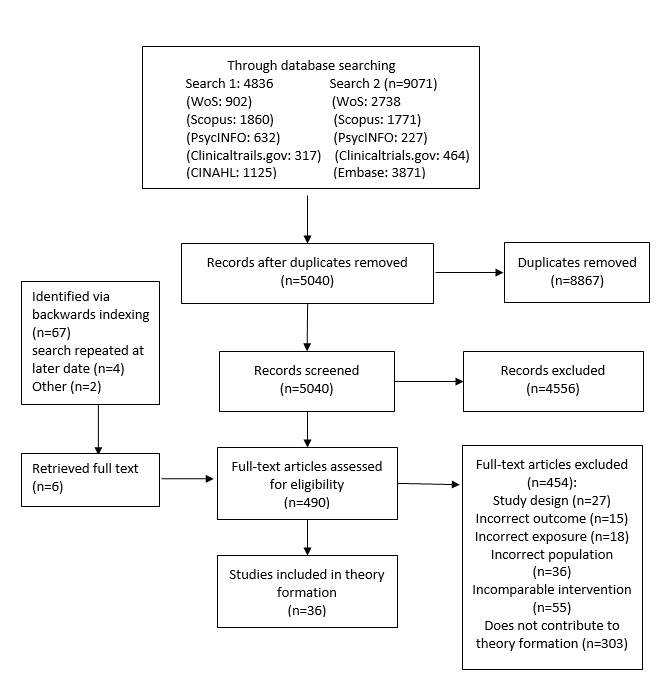


Figure 1: PRISMA Flow Diagram - articles retrieved from CINAHL, Embase (Search 2 only), Scopus, Web of Science, Clinicaltrials.org and PSYCHinfo (Search 1 only)
