# Supplementary material for: Plant-based diets for older adults in care homes: a realist synthesis
Source: BMC Geriatr. 2026 Jan 26;26:233. doi: 10.1186/s12877-025-06927-0 (PMC12918464; doi:10.1186/s12877-025-06927-0)
Supplement: Supplementary file 5 — Additional file 5. Study set information: generic information on the studies used in this realist synthesis. [file 12877_2025_6927_MOESM5_ESM.docx]

| **Lead Author and Date** | **Study Design** | **N=** | **Participant Characteristics** | **Country** | **Length of study period** | **Diet type** | **Method** | **Outcome Measures** | **Study Quality** |
| --- | --- | --- | --- | --- | --- | --- | --- | --- | --- |
| Bailey (2018) | Qualitative | 6 | Aged 72-89, length of stay: 6 months-7 years, 83.3% female | Australia | N/A |  | Semi-structured Interviews | What impacts meal liking, understanding of policy, food literacy | High |
| Brown (2012) | Qualitative | 17 | 88.2% females, employed for 0.5-30 years | USA | N/A | PBD | Interviews | Self-efficacy, productivity, job satisfaction, wellness | High |
| Cave (2021) | Qualitative | 21 | Foodservice  managers, foodservice workers, dietitians, carers, and other managers | Australia | N/A |  | Semi-structured interviews | Impact of resident input on menus | High |
| Crogan (2013) | Quasi-experimental | 9 | 1 man in 20s, 2 women in 40s, 1 man in 50s, rest older. 6 women, 3 men | USA | 6 months |  | Two arm control trial - Impact of increasing control over food | Resident satisfaction, food intake and nutritional status | High |
| Drolet-Labelle (2023) | Qualitative | 60 | 65+, 40 non-plant protein consumers, 40 consumers | Canada | N/A |  | Semi-structured interviews | Pros and cons to consume plant-based protein | High |
| Ewens (2023) | Quasi-experimental | 194 | 76.3% female, 28.9% HCPs. 63.4% meat eaters, between 25-34 years old | New Zealand | N/A | Meat-free | Meat-free Mondays provided | Awareness, support for intervention, barriers, enablers | High |
| Grasso (2021) | Quantitative | 2500 | 65+, community-dwelling | Poland, Finland, Spain, Netherlands, UK | N/A | PBD | Survey | Reasons for choosing to consume or not consume meat | High |
| Hoefnagals (2023) | Qualitative | 32 | food service staff members (n = 16), comprising of kitchen staff (n = 4), wait staff (n = 10), and facility management (n = 2) | Netherlands | N/A |  | Semi-structured Interviews | Opinions on sustainability in care homes | High |
| Shune (2022) | Qualitative | 10 | 55-89 years old (MAge 69.7 +/- 12.8), 80% female, 50% dysphagic, 40% of textured modified diets | USA | N/A |  | Semi-structured Interviews | What influences eating and food intake | High |
| Watkins (2017) | Qualitative | 11 | 6 women, 5 men, MAge=87 (78-97) | UK | N/A |  | Semi-structured Interviews | What impacts the mealtime experience | High |
| Wheeler (2025b) | Qualitative | 20 | 12 female, 9 between 65-74 | Australia | 6 weeks |  | Interviews | Food choice needs in care homes | High |
| Bamford (2012) | Qualitative, Process evaluation | 112 | 25-40 bed homes | UK | N/A |  | Observations, Interviews | Nutritional guidelines and how they can fit into care home menus | Moderate |
| Bianchi (2022) | RCT | 115 | Aged mid-20s- mid-40s, 60% female, majority had a college degree, 50% lived alone | UK | 4 weeks | Meat-free | Intervention, participants given free meat substitutes, vignettes of success stories, information leaflets | Positive attitudes, perceived control, subjective norms of eating a low meat diet, attachment to meat, weight loss, intentions to change | Moderate |
| Chwyl (2024) | Quasi-experimental | 20 | MAge 54.3 (36-71), overweight or obese, 100% female, 80% white | USA | 12 weeks | PBD | Intervention including Leadership training and coaching, organisation culture, risk awareness, incentives, social support | Experiences on programmes, enablers and hindrances to adherence, perceived benefits | Moderate |
| Davies (2022) | Qualitative (Ethnography) | 250 | 3 care units | UK, Netherlands, Switzerland |  |  | Observation, interviews, informal conversations | What influences mealtime enjoyment | Moderate |
| Dogra (2020) | Quasi-experimental | 18 | Not stated | USA | 8 weeks | PBD | provision of weekly groceries, two-week sample menu, vegan/AHA cookbook, measuring tools, and at least twice weekly contact with a registered dietitian | Weight loss, barriers and facilitators to adherence | Moderate |
| Ducak (2011) | Qualitative | 40 | 5 dieticians, 40 nutrition managers | Canaca | 7-day menu |  | Development and costing of hypothetical menu, semi-structured interviews | Influence on nutrition intake | Moderate |
| Duizer (2020) | Qualitative | 13 | 7 dieticians, 6 nutrition managers | Canada | N/A |  | Telephone Interview | Meal planning | Moderate |
| KejZar (2022) | Mixed methods | 102 | 87.5% for the congruent  care group and 62.5% for the non-congruent group. Total n=12 care homes | Slovenia | N/A |  | Interviews, Quantitative comparative analysis | How congruency affects meal planning and quality of life | Moderate |
| Mahadevan (2014) | Qualitative | 38 | Age 65+, male (n=13), females (n=25) | USA | N/A |  | Focus groups | Mealtime experience | Moderate |
| Matwiejczyk (2018) | Qualitative | 31 | Senior level chefs | Australia | 4 months |  | Pre-post-test questionnaires, focus groups | Knowledge of staff, opinions on programme | Moderate |
| Milte (2017) | Qualitative | 25 | n=6 family members, n=13 residents living with CI, n=3 community dwelling, n=3 nursing home without CI | Australia | N/A |  | Interviews, focus groups | What influences mealtime enjoyment | Moderate |
| Morin (2019) | Quasi-experimental | 72 | Mainly women, aged 45+, 30% on cholesterol reducing drugs | Canada | 12 weeks | Whole food PB | Workshops, practical classes | Habits and changes to diet in response to programme | Moderate |
| Murphy (2017) | Qualitative | 50 | nurses, care workers, catering assistants, dietitians, speech and language therapists, and family carers. | UK | N/A |  | semi-structured interviews, focus groups | What increases food intake | Moderate |
| Neilsen (2025) | Qualitative | 20 | Length of stay: 2 months-5 years, aged 78-96, 13 women, 7 men, 50% in dementia care | Sweden | N/A |  | Semi-structured interviews | Explore residents’ experiences of mealtimes | Moderate |
| Okpara (2022) | Qualitative | 84 | Most BMI between 25-49.9, African Americans | USA | N/A | PBD | Focus groups | Understanding food preferences and how it relates to identity | Moderate |
| Tsai (2020) | Qualitative | 18 | MAge= 62.72, 56% female, most married. NH residents were mean age 86.27, 61.1% female, 22.12 months of residency | Tiawan | N/A |  | Face to face interviews | Reasons for visiting loved ones during mealtimes | Moderate |
| Van Wymelbeke (2020) | Quantitative (Control trial) | 82 | 63 women, 19 men, MAge 87.5 (between 71-101) | France | N/A |  | Providing new meal options | Impact on liking and intake | Moderate |
| Wheeler (2025a) | Quasi-experimental | 14 | 12 female, 9 between 65-74 | Australia | 6 weeks |  | Design and evaluation of an intervention | Food waste, resident satisfaction, food service costs | Moderate |
| Albert (2022) | Quasi-experimental | 109 | female (67.0%), average 53 years old, 39.0% Black/African American, 31.4% White | USA | 12 months |  | Intervention with PB education, individual consultations, nutrition plan, starter guide, cookbook, regular check ups | Participant motivation, what motivated them to participate, qualitative analysis on opinions | Low |
| Amiot (2018) | RCT | 32 | Male, aged 18-30, 18-29 BMI, free from chronic disease | Canada | 4 weeks | Meat free | Education intervention with goal setting, monitoring with participants keeping a journal | Attitudinal and emotional changes, ability to stick to goals | Low |
| Carrier (2007) | Qualitative | 395 | 38 NH, 65+, 66.5% were cognitively impaired, 26.8% of whom had a BMI less than 20kg/m2 compared to those who were cognitively intact (9.1%). | Canada | N/A |  | Interviews, Observations | Dining experience, quality of life | Low |
| Singh (2020) | Mixed methods | 98 | 87% females, 93% overweight | USA | 6 weeks, 3 months maintenance |  | Culturally tailored plant-based education | Adiposity parameters, opinions of programme | Low |
| Stiles (2024) | Qualitative | 35 | foodservice dietitians n = 10; foodservice managers, n =6; dietetic  professional leads n = 4; chef n = 4; information technology n = 4; contract manager n = 4; other manager n = 3 | New Zealand | N/A | PBD | Interviews | Benefits and risks of providing PB in hospitals | Low |
| Karlsen (2017) | Opinion piece | N/A | N/A | USA | N/A | PBD | N/A | Motivational interviewing and other strategies to increase PB food uptake | N/A |
| Salvidar (2021 | Evaluation | N/A | N/A | USA | N/A | PBD | N/A | Concerns and future goals with implementing PBMs into hospitals | N/A |
