# Supplementary material for: Plant-based diets for older adults in care homes: a realist synthesis
Source: BMC Geriatr. 2026 Jan 26;26:233. doi: 10.1186/s12877-025-06927-0 (PMC12918464; doi:10.1186/s12877-025-06927-0)
Supplement: Supplementary file 6 — Additional file 6. Building of theories: process of building initial programme theories from retrieved literature. [file 12877_2025_6927_MOESM6_ESM.docx]

| Author (Year) | Title | Relevance | Contexts | Intervention Activity | Mechanism | Outcome |
| --- | --- | --- | --- | --- | --- | --- |
| Albert (2022) | Pilot Plant-Based Lifestyle Medicine Program in an Urban Public Healthcare System: Evaluating Demand and Implementation | High |  | Intervention guided by someone with expertise and experience (+), Building relationship with intervention leader (+), Flexibility of adherence (+), Individualisation of intervention (+), Resources to educate (+) | Increased trust (+), Increased accountability (+), Increased Reflexive Motivation (+) | Increased confidence in participation (+), more likely to adhere to intervention (+), Increased Access |
| Bamford (2012) | Implementing nutrition guidelines for older people in residential care homes: a qualitative study using Normalization Process Theory | High | Health-conscious (+), Proud of existing menu (-), Poor communication between staff and managers (-), Supporting management team (+), open to learning new skills (+), focus on homely environment (-), Poor understanding of healthy eating (-), open to change (+), Positive attitude (+) (Staff) | Lack of feedback from entire team (-), whole team engagement (+), Cooks involved in menu development (+), adaption of traditional meals (+), Lack of resources to implement (-), Access to knowledgeable research team (+) | Perception of intervention as ‘extreme’ (-), Perception that intervention incompatible with home (-), Perceive negative reactions from residents (-), Perceived threat to autonomy (-), lack of reflexive motivation (-), Feel ownership over menus (+), conflicts with personal (chefs) preference (-), Lack of confidence in intervention (-) | Lack of intervention adherence (-), Refusal to implement (-), Residents resistant to change (-) |
| Cave (2021) | Food and Nutrition Champions in Residential Aged Care  Homes Are Key for Sustainable Systems Change within  Foodservices; Results from a Qualitative Study of Stakeholders | High | Understand importance of food for health (Staff) (+), Residents who desire autonomy over food (+), Poor staff attitude towards intervention (-), CH culture of continuous improvement (+),CH manager on board with intervention (+), Positive attitude to investing in staff training (+) | Tasty menu items (+), resident feedback to adapt meals (+), Greater choice (+), Collaboration between all staff (+), Knowledgeable intervention leader (+), Education (+), Menu tailored to fit budget of CH (+) | Automatic motivation (+), Physical opportunity (+), Greater sense of autonomy (Residents) (+), Increased psychological capability (+), Increased understanding of importance (+), Perceive change as easier (+) | Increase likelihood of choice (+), Greater consumption of food (+), Feel valued and respected by staff (+), Better implementation of intervention (+) |
| Crogan (2013) | Food choice can improve nursing home resident meal service satisfaction and nutritional status | High |  | Increasing access to diverse foods (+), Improved food quality (+), Incorporation of resident opinions into meal development | Increased autonomy (+), Greater perceived control (+) | Improved resident satisfaction with mealtimes (+) |
| Drolet-Labelle (2022) | Beliefs underlying older adults’ intention to consume plant-based protein foods: A qualitative study | High | Residents previously familiar with PB foods (+), Staff who perceive PB foods as unhealthy (-), Residents perceiving PB meals to be not tasty (-), Residents open to trying new foods (+), Residents lacking motivation to change (-) | Increase familiarity via tasting sessions (+), Meal preparation training (-), Support from staff (+), Increased access (+), Reorganisation of menu to promote PB foods (+) | Increased physical opportunity (+), Increased psychological capability (+), increased perceived social acceptability, Increased reflexive motivation (+) | Improved perception of PB meals (+), increased choice of PB meals |
| Hoefnagals (2023) | The perceptions of food service staff in a nursing home on an upcoming transition towards a healthy and sustainable food environment: a qualitative study | High | Poor communication between staff and managers in CH (-), Staff not focused on improving health (-), Staff open to new ideas (+), Staff perceive residents will react negatively (-) | Inter-professional involvement in meal development (+), Staff education (+), Meal training for chefs (+), Meal tasting sessions (+) | Increased perception of physical capability (+), Greater trust in own and other abilities (+) | Increased confidence in intervention (+) |
| Matwiejczyk (2018) | Engaging food service providers to change food service practices in aged care facilities | High | Motivated and willing chefs (+) | Meal training for chefs (+) | Increased physical capability (+) | Increased understanding of how to prepare good PB food (+), Better dining experience (+) |
| Saldivar (2021) | Successful Incorporation of a Plant-Based Menu Into a Large Academic Hospital | High |  | Staff try meals initially and chose most palatable (+), option to provide meat-based meal (+), PB menu items cheaper (+) |  | Satisfaction with plant-based meals (+) |
| Stiles (2023) | Increasing the proportion of plant to animal protein in hospital patient menus: what do stakeholders think? | High | Staff believe intervention unfeasible (-), Believe residents will not accept meals (+) | Adapt to budget of CH (+), Lower cost health swaps (+), Maintaining choice over meals (+), reorchestrating menu to promote PB foods (+) | Automatic motivation (+) |  |
| Watkins (2017) | Exploring residents’ experiences of  mealtimes in care homes: A qualitative  interview study | High | Residents who only like familiar foods (-), CHs with limited resources (+) | Culturally-familiar foods (+), increased choice, feedback from food tasting sessions (+) | Connection to previous routines (+) | Increased comfort and homeliness of CH (+) |
| Wheeler (2025a) | Choice on the menu: Increasing meal choice for people living in residential aged care, a pilot study 49 | High |  | Feedback from tasting sessions (+), inter-professional collaboration from all staff (+), Improved choices (+) |  | Increased fruit and vegetable consumption (+), Increased food service satisfaction |
| Wheeler (2025b) | Choice on the menu in residential aged care: An underrated  tool for maintaining resident autonomy 41 | High | Poor collaboration in CH (-) | Improved choices (+), improved chef food prep skills (+), high quality ingredients, feedback through food tasting sessions (+), opportunity to voice opinions through intervention (+) | Greater sense of autonomy (+), increased empowerment | More comfort and value derived in CH setting (+), Increased meal enjoyment |
| Brown (2021) | Constructing a Grounded Theory of Worker Attitudes Toward Developing a Plant-Based Nutrition Intervention 34 | Moderate | Organisations’ structure supports change (+), Indifference towards meat (Residents) (+), Life course heavily influenced by meat (Residents/Staff) (-) | Education (+), awareness across staffing team (+), Support from staff (+), Provide rationale for participation (+), Financial incentive (+), Support from others (+), Increased availability of PB option (+), Flexibility of choice (+), Support from managers (+) | Increase psychological capability (+), Increased reflexive motivation (+), Increase belief in feasibility (+), Changed perception on importance of meat (+) | Greater intervention adherence (+), Increased PB choice (+) |
| Carrier (2009) | Dining experience, foodservices and staffing are associated with quality of life in elderly nursing home residents 54 | Moderate | Desire greater autonomy over food choice (residents) (+) | Greater food choice (+) | Feelings of autonomy over choice (+) | Greater quality of life |
| Chwyl (2024) | “Mindset Matters”: Perseverance, a balanced approach and structured support as facilitators of whole foods plant-based adoption 36 | Moderate | Positive attitude towards intervention (+), Lack of familiarity and experience with PBD (-), Open-minded (Staff/Residents) (+), Open to new foods (Residents) (+) | Flexibility in choice (+), Adapting traditional meals (+), easy to prepare meals (+), Individualisation (+), Education (+), Presence of intervention leader (+), Availability/convenience of PB meals (+), Cooking training (+) | Increase psychological capability (+), Increased sense of accountability (+), Change in attitudes towards PB meals (+) | Greater adoption of intervention (+), Feel valued (Residents) (+), Feel better after meal consumption (+) |
| Davies (2022) | Exploring resident experiences of person‑centred care at mealtimes  in long‑term residential care: a rapid ethnography 16 | Moderate | CHs that can support person centered care (+) | Offering greater choice for those with dietary requirements (+), incorporation of resident opinions into meal development (+) | Increased provision of person-centered care (+) | More positive view of CH meals and staff (+), Improved quality of life |
| Ducak (2011) | Menu Planning in Long-Term Care:  Toward Resident-centred Menus 29 | Moderate | CH lacks resources to meet resident’s needs (-), Multi-cultural homes (+) | Incorporate resident feedback into meals (+), Increased choice (+), Access to specialised PB food items (+), use of staple food products (+) |  | Improved food intake (+) |
| Duizer (2021) | Planning Micronutrient-Dense Menus in Ontario Long-Term Care Homes: Strategies  and Challenges 30 | Moderate | Multi-cultural/diverse CHs (+), CHs with financial limitations (+) | Ensuring meals are tailorable to CH needs (+), Ensuring meals are familiar and meet preferences (+), Addition of PB swaps (+) |  |  |
| Ewens (2023) | Meat-Free Mondays in Hospital Cafés in Aotearoa, New Zealand 44 | Moderate | Previous familiarity with PB meals (+), CHs driven by health (+), Attitude that meat is a necessity (-), residents/staff open to new foods (+) | Do not limit choice of meat meals (+), delicious PB meals (+), Staff education (+) |  | Increased support for intervention (+) |
| Grasso (2021) | Understanding meat consumption in later life: A segmentation of older consumers in the EU | Moderate | Residents who consume lower amounts of meat (+), Residents with high liking of meat (-), Residents with a smaller appetite (+), Residents who are fussier over food (-) |  |  |  |
| Karlsen (2017) | Strategies for practitioners to support patients in plant-based eating 35 | Moderate | Residents open to new flavours (+) | Education for staff (+), Lower cost alternatives (+), Staple foods (+) | Increase positive perception towards intervention (+), Increased self-efficacy/reflexive motivation (+) | More likely to adhere to meals (+) |
| Mahadevan (2014) | Assisted-living elderly and the mealtime experience | Moderate |  | Attractive meal presentation (+), Increase menu choice (+), Familiar traditional meals (+) | Automatic motivation (+), Increase perceived control (+) | Improved perception of mealtimes (+), Improved well-being (+) |
| Milte (2017) | Struggling to maintain individuality – Describing the experience of food in nursing homes for people with dementia | Moderate |  | Feedback from meal tasting sessions (+), Greater choice (+) | Increased perceived control (+) | Better quality of life (+) |
| Murphy (2017) | Nutrition and dementia care: developing an  evidence-based model for nutritional care in nursing homes | Moderate | Inadequate time and financial resources (-) | Education for staff (+), feedback through food tastings (+), greater choices |  | Improved provision of meals (+), better support of different cultures (+) |
| Nielsen (2025) | Eating Together but Often Feeling Lonely: Residents' Mealtime Experiences in a Nursing Home | Moderate |  | Familiar, traditional meals (+), Meals that don’t meet textural needs (-) | Preconceived idea of how food will taste leading to automatic motivation (+) | Increased meal enjoyment (+) |
| Okpara (2021) | “Food Doesn’t Have Power Over Me Anymore!” Self-Efficacy as a Driver for Dietary Adherence Among African American Adults Participating in  Plant-Based and Meat-Reduced Dietary Interventions: A Qualitative Study 42 | Moderate | Residents who desire nostalgia and comfort from food (+) | Tasting sessions for residents and staff (+), meal preparation for chefs (+), emphasis on traditional meals (+), elimination of meat based meals (-) |  | Increased positive perception of PB meals |
| Tsai (2019) | “Tailoring homely meals”: Family members’ motivations  underlying nursing home visits during residents’ meals 50 | Moderate |  | Tailoring to cultural needs (+), Co-design with chefs to ensure foods are delicious (+), Increase variety (+) |  | Making mealtime more homelike (+), Increased meal intake (+) |
| Van Wymelbeke (2020) | Optimizing sensory quality and variety: An effective strategy for increasing  meal enjoyment and food intake in older nursing home residents 20 | Moderate |  | Increased food quality (+), Increased variety (+) |  | Increased meal enjoyment (+), Increased food consumption (+) |
| Amiot (2018) | Testing a novel multicomponent intervention to reduce meat consumption in young men 45 | Low | Positive emotions towards meat (-), indifference towards eating meat (+) (Residents) | Education to build rationale for taking part (+), autonomy of choice (+) | Increased Reflexive Motivation (+), Increased psychological capability (+) | Reduced belief in the importance of providing meat-based dishes (+) |
| Bailey (2017) | ‘I’d rather die happy’: residents’ experiences with food regulations, risk and  food choice in residential aged care. A qualitative study 55 | Low | Desire autonomy over choice (+) | Individualisation of intervention (+), fresh food (+), no restriction on choice (+), |  | Greater meal enjoyment (+) |
| Bianchi (2021) | Replacing meat with alternative plant-based products (RE-MAP): a randomized controlled trial of a multicomponent behavioral intervention to reduce meat consumption 58 | Low |  | Tasting products (staff/residents) (+) | Increase positive attitude (+), Decreased attachment to meat (+) | Reduced consumption of meat-based meals (+), Improvement in blood pressure (for those adherent) (+) |
| Dogra (2022) | Long-term Dietary and Weight Changes Following a Short-Term Dietary Intervention Study: EVADE CAD Trial Follow-Up 43 | Low | Chefs lacking in PB meal preparation skills (-), Residents with preference for meat (-) | Support from staff (+), PB options more convenient to choose (+) | Increased perceived physical opportunity (+) | Improved quality of life (+) |
| Kejzar (2022) | Nutrition and Congruent Care  Improve Wellbeing of Residents With  Dementia in Slovenian Care Homes 56 | Low |  | Education for staff (+), feedback obtained through meal tasting (+), greater food choice (+) |  | Improved autonomy (+) |
| Morin (2019) | A whole-food, plant-based nutrition program: Evaluation of cardiovascular outcomes and exploration of food choices determinants 46 | Low |  | Education for staff (+) | Shift in values towards health (+) | Increased advocation for PB meals (+) |
| Shune (2022) | Redefining the value of snacks for nursing home residents: Bridging psychosocial and nutritional needs 47 | Low |  | Tasty, visually appealing meals (+) Traditional foods (+), Staff education | Automatic motivation (+), Increased reminiscence (+) | Increased meal enjoyment (+) |
| Singh (2020) | Incorporating an Increase in Plant-Based Food Choices into a Model of Culturally Responsive  Care for Hispanic/Latino Children and Adults  Who Are Overweight/Obese 33 | Low |  | Education for staff (+), culturally tailored meals (+), Financial support (+) | Perceived psychological capability (+) | Increase meal choice (+), increased meal acceptance (+) |
